# Storage Stability of Durum Wheat Pasta Enriched with Seaweeds Flours

**DOI:** 10.3390/foods10102450

**Published:** 2021-10-14

**Authors:** Ana Ramalho Ribeiro, Goreti Botelho, Ana Gaspar, Rui Costa

**Affiliations:** 1 Polytechnic Institute of Coimbra, Coimbra Agriculture School, Bencanta, 3045-601 Coimbra, Portugal; anarribeiro@gmail.com (A.R.R.); goreti@esac.pt (G.B.); anafbgaspar@gmail.com (A.G.); 2 Research Centre for Natural Resources, Environment and Society (CERNAS), Coimbra Agriculture School, Bencanta, 3045-601 Coimbra, Portugal

**Keywords:** storage stability, shelf life, pasta, seaweeds, sensory acceptance, cooking quality

## Abstract

The enrichment of semolina pasta with nutritionally rich ingredients has been targeted as a health strategy in recent years. In this work, the storage stability of seaweed-enriched pasta was assessed at different combinations of temperature and relative humidity. After six months of storage, pasta samples did not present variations in their sensory properties. The enrichment of durum wheat pasta with 1% of macroalgae *Fucus vesiculosus* and *Ulva rigida* flours, or flours of its extracts, was found to be adequate without influencing or modifying the sensory characteristics of pasta samples during the storage period. Water activity was shown to be the main criteria influencing the quality parameters of pasta during shelf life. A higher water activity during storage will lead to higher cooking losses and a lower firmness of cooked pasta, which will damage pasta quality over time.

## 1. Introduction

Dried pasta is one of the most successful convenience manufactured food products in modern society. Classic pasta is prepared with durum wheat semolina, mixed with water, kneaded, extruded into many possible different shapes, and dried [1]. Pasta is served in a great variety of ways, especially due to its property of retaining the flavor and taste from other ingredients, enlarging its culinary possibilities [2]. This characteristic also favors pasta as a fortification medium for different nutrients to prevent malnutrition, as suggested by the Food Agriculture Organization and the World Health Organization [3]. As a consequence, pasta enrichment is the subject of studies with the incorporation of legumes, other cereals, dairy by-products, and yeast, among others [4]. Pasta consumption is increasing according to a recent study which indicated that more than 90% of the consumers in France, Germany, UK, and USA eat pasta, and the average per capita consumption varies between 3.5 kg in the UK and 23.5 kg in Italy [5]. 

The use of algae as food ingredients has increased due to its health benefits [6]. In particular, seaweeds are associated with a reduced incidence of chronic diseases [7]. Some micronutrients and bioactives are present in such a high amount that a small incorporation in another product can make a difference. For example, a dried extract from *Fucus vesiculosus* incorporated in pasta at a 1% level could supply enough iodine for one day in 100 g of pasta [8].

Durum wheat pasta is a shelf stable product that can maintain the same properties for long periods of time. This empirical knowledge led to the near absence of studies on the shelf life of semolina dry pasta. However, the constant innovation trend to supply the food market with new and healthier products, tailored to the increasing diversity of consumer profiles, led to the development of durum wheat pasta enriched with many functional ingredients such as protein, dietary fiber, pigments or antioxidants, among others [9], and the enrichment strategy referred above. These new products need an assessment of the shelf life prior going to the market and this process is the subject of many published works, such as pasta fortified with legumes or protein concentrates [10] or with polyunsaturated fatty acids [11]. 

The storage of food products occur in a wide range of environmental conditions. Ambient conditions are usually recommended for dry-packed products. However, a wide range of temperature and relative humidity conditions exist in different regions of the world which will inevitably lead to water losses or gains in hygroscopic products such as pasta, even when packed. The accelerated tests of shelf life are used to decrease the time needed to estimate the shelf life of products with an expected long shelf life, such as dry products. Changing environmental conditions that affect the deteriorative reactions, is the strategy to accelerate the tests. The temperature is the most common environmental factor to use in accelerated tests [12] and, regarding pasta, relative humidity should also be considered because it affects the water activity of the pasta, a crucial parameter that affects chemical and microbiological reactions.

To the authors’ knowledge, no storage stability study of enriched durum wheat pasta with seaweeds has been conducted, nor has another shelf-life study of dry pasta assessed water activity as the main experimental parameter. This study aimed to assess the influence of seaweeds flour from *Fucus vesiculosus* and *Ulva rigida* and its corresponding water-based extracts on the storage stability of durum wheat pasta. *Fucus vesiculosus* and *Ulva rigida* are typical seaweeds of the Portuguese coastal area with a sustainable production, and their inclusion in food products is being promoted as they are functional ingredients to promote health and increase food quality. Both seaweed flours and dried extracts were incorporated in pasta at a 1% (g/100 g) level, a concentration adopted by industrial dry pasta produced on a large scale with ingredients such as tomato or cuttlefish ink. This was now explained in the introduction section. During the stability trial, pasta samples were stored at different equilibrium water activities controlled by temperatures between 5 and 30 °C with a relative humidity varying between 50 and 65%, by studying the effect on cooking and sensory properties.

## 2. Materials and Methods

### 2.1. Ingredients

Durum wheat semolina (DWS) was purchased from Cerealis—Produtos Alimentares, S.A. (Maia, Portugal). Dried and milled *Fucus vesiculosus* and *Ulva rigida* flours (<0.25 mm) were supplied by ALGAplus—Produção e Comercialização de Algas e seus Derivados (Ílhavo, Portugal). 

Extracts of both seaweeds were prepared by using 140 mL of distilled water (pH = 6.6 at 25 °C) per 7 g of seaweed flour, in Duran flasks at 120 °C heated in a retort (Raypa AES-75, Barcelona, Spain) for 2 h. The solubilized material was separated from the sediments in a centrifuge at 20 °C and 6780 rpm for 10 min, followed by freezing and freeze-drying (UNICRYO MC-4L-60 °C, Martinsried, Germany). The resulting dried extracts were kept frozen until pasta production.

### 2.2. Pasta Production

Four experimental pasta samples and one control pasta were used in the storage stability tests. The control pasta was composed of 100% of durum wheat semolina (DWS). Experimental pastas were prepared with durum wheat semolina and seaweed flours or its extracts at 1% incorporation. Flours of *Fucus vesiculosus* (F) and *Ulva rigida* (U), and extracts of *Fucus vesiculosus* (eF), and of *Ulva rigida* (eU), were added as 1 g of seaweed flour per 100 g of mixture. 

After homogenization of DWS and seaweed flour, each mixture was mixed with adjusted levels of water of 40, 40, 41, 42 and 42 mL of water/100 g mixture, respectively, for DWS, eU, F, eF and U, as determined for each formulation by previous trials. The blends were mixed and kneaded (Philips Avance Collection HR2354/12, Amsterdam, The Netherlands) into a homogenous dough and left to rest for 10 min. The dough was extruded as lasagna sheets with a fresh pasta Maker Machine (Philips Avance Collection HR2354/12, Amsterdam, The Netherlands) and cut as fettuccine with a manual pasta machine (ITALIA, Casa International NV, Olen, Belgium). The experimental pasta was dried in a ventilated oven (Falc Oven Model STE-F 52, Treviglio, Italy) at 55 °C until a moisture level of approximately 11% was reached.

After cooling, the pasta was packaged with oriented polypropylene (OPP) film and heat-sealed using a hand sealing machine. The OPP film with a water vapor transmission rate (WVTR) < 3.0 g/m^2^/24 h/atm determined at 38 °C-90% RH (Method ASTM F1249), was kindly supplied by the company Monteiro Packaging (Porto, Portugal).

### 2.3. Storage Stability Tests

The packaged pastas were stored in four conditions of storage: 5 °C-50% RH, 20 °C-55% RH, 20 °C-65% RH and 30 °C-50% RH. Chambers with controlled temperature and humidity were used for the 5 °C-50%RH, 20 °C-65% RH and 30 °C-50% RH conditions. Pasta samples in the 20 °C-55% RH condition were kept at a room temperature storage in a laboratory room with controlled temperature. These conditions enabled the testing of the influence of temperature and water activity on pasta properties. The lowest temperature was chosen as the condition where chemical reactions would present very low rates and the product characteristics would be kept constant. 

Sensory and physical chemical analyses were performed after a reversed storage design [12] to have all the samples available for sensory analyses on the same day. This design allowed a better comparison between samples. The samples from all experimental pastas were manufactured in the same week and stored at a considered control condition of 5 °C-50% RH. Sequentially pasta samples were moved into the test conditions after 2 months, corresponding to 4 months in test conditions; or after 4 months, corresponding to 2 months of storage at the different test conditions (Table 1). The samples prepared for 6 months of storage were immediately conserved at the selected test conditions. For example, samples to be tested with 4 months of storage at 30 °C-50% RH were kept at the lowest temperature for 2 months before being placed at the required conditions of 30 °C-50% RH. Control samples (CTRL) were kept at 5 °C-50% RH for 6 months. 

### 2.4. Sensory Analysis

Sensory analysis of the pasta stored at controlled humidity and temperature conditions was performed using the Difference From Control Test [13]. This test was used to determine whether there was a noticeable general difference between one or more samples and a control sample and could also indicate the magnitude of the perceived difference. 

Ten sensory tests were carried out comparing pasta maintained in conditions of 5 °C-50%RH for six months (CTRL), with pasta preserved for two (M2), four (M4), and six (M6) months at 20 °C-55% RH, 20 °C-65% RH and 30 °C-50% RH. 

Seven tasters participated in each test, of which four participated in all tests and three were variable tasters from a panel of a total of fourteen tasters. Written informed consent was obtained from all volunteers. The sensory tests took place in an acclimatized room with individual test cabinets (ISO8589 [14]). For each session, seven samples were prepared per taster: an identified control and six samples of pasta with seaweed or pasta with extract, differing in temperature and storage time. For example, CTRL pasta was compared with the same pasta stored at 20 °C-50% RH during 2, 4, and 6 months and with samples stored at 30 °C-50% RH during 2, 4, and 6 months. Each experimental pasta was cooked to its optimal cooking time and immediately placed in white cups and covered. The samples, encoded with three digits, were presented at random after the CTRL condition sample. The tasters were asked to rate the difference from the control on a linear scale from zero (no difference to the control) to 12 cm (totally different from the control). The results obtained were analyzed with one-way ANOVA and compared using the Tukey test. Statistical significance was tested with a probability level of 0.05.

### 2.5. Physicochemical Analysis

The following physicochemical analyses were carried out at the optimal cooking time. Optimal cooking time was determined when the central white core disappeared when squeezed between two glass plates. Optimal cooking times (OCT) did not vary between the months during storage and were 9 min for DWS, 7 min for eU, 10 min for eF and U, and 13 min for F. The cooked pasta was transferred into a beaker with distilled water at room temperature before testing. All tests were performed in triplicate.

Samples were analysed after pasta production, before the beginning of storage trial and after six months under testing conditions of controlled temperature and relative humidity.

#### 2.5.1. Weight Gain

Weight gain was determined as the increase in pasta weight over the dry pasta weight:

(1) $$\mathrm{Weight}\;\mathrm{gain}=\frac{\mathrm{weigth}\;\mathrm{of}\;\mathrm{cooked}\;\mathrm{pasta}\;(g)\;-\mathrm{weight}\;\mathrm{of}\;\mathrm{dry}\;\mathrm{pasta}\;(g)}{\mathrm{weight}\;\mathrm{of}\;\mathrm{dry}\;\mathrm{pasta}\;(g)}$$

#### 2.5.2. Cooking Loss

Cooking loss was evaluated by determining the mass of solids lost into cooking water [15]. After cooking, each sample was rinsed with 50 mL of filtered water in a Buchner funnel and drained for 2 min before weighing. Cooking and rinsing water were recovered. The material leached to the cooking water was determined by evaporating the cooking water and rinsing water until dry in an air oven at 105 °C. The residue was weighed and reported as a percentage of the original pasta sample according to AACC standard 66–50. All tests were performed in duplicate.

Cooking loss was determined as:

(2) $$\mathrm{Cooking}\;\mathrm{loss}=\frac{\mathrm{weight}\;\mathrm{of}\;\mathrm{cooking}\;\mathrm{water}\;\mathrm{residue}\;(g)}{\mathrm{weight}\;\mathrm{of}\;\mathrm{dry}\;\mathrm{pasta}\;(g)}\;\times \;100$$

### 2.6. Texture Analysis

The texture of raw and cooked pasta was determined using a TAXT Express Texture Analyzer equipped with a Windows version of Texture Expert software package (Stable Micro Systems, Surrey, UK). Uncooked pasta firmness was measured using a three-point bend test. A single pasta strand with 5 cm length was placed perpendicular to two 11 cm triangular perplex supports, which were fixed in a perplex base and distanced by 4 cm between the tops and cut with a light knife blade attachment (thickness 1 mm). Test was repeated with new pasta strands at least five times. The maximum force value was recorded as raw pasta firmness.

Testing of cooked pasta was performed immediately after cooking to minimize changes resulting from being preserved in a liquid medium. Cooked firmness was determined according to AACC standard 66–50. Four fettuccine shaped pasta strands were placed in the center of the measuring area and cut with a light knife blade attachment (thickness 1 mm, A/LKB Stable Micro Systems, Ltd., Surrey, UK). The maximum force value was recorded as pasta firmness (g). Test parameters were 2 mm/s pre-test speed, 3.0 mm/s test speed, 10 mm/s post-test speed; distance was adjusted to a maximum of 5 mm, 15 g was fixed as trigger force, and 5 kg of load cell mass was used. 

### 2.7. Water Activity

The water activity of dry pasta was performed using a Rotronic Hygrolab c1 device at 20 ± 1 °C. Measurements were performed, at least in duplicate, until a constant value was reached.

### 2.8. Pasta Isotherm Data

The estimations of water content (X, g/100 g of solids) and water activity based on the storage conditions of the trials were calculated using the Oswin model and respective constants were presented by De Temmerman et al. [16]:

(3) $$X=\left(A_{1}-A_{2}T\right){\left(\frac{1-a_{w}}{a_{w}}\right)}^{\left(B_{1}-B_{2}T\right)}$$

A_1_ = 0.138

A_2_ = 10.4 × 10^−4^

B_1_ = 0.396

B_2_ = 11.6 × 10^−4^

### 2.9. Statistical Analysis

Data relative to instrumental determination of cooking quality were subjected to an analysis of variance using the IBM SPSS Statistics (v25, IBM, Armonk, NY, USA). ANOVA was followed by Tukey post hoc test. Non-parametric Kruskal–Wallis test was used when experimental data did not meet ANOVA assumptions. Significance level used was *p* < 0.05.

## 3. Results and Discussion

### 3.1. Water Activity of Pasta

Hygroscopic products, such as pasta, absorb or loose water when exposed to an ambient relative humidity which is above or below their relative humidity at equilibrium (i.e., its water activity). For example, a product with a water activity of 0.5 at a temperature T will suffer no gains or losses of water when exposed to air with a relative humidity of 50% at the same temperature T. However, when exposed to a different relative humidity at the same temperature T, the product will lose or gain water if the relative humidity is lower or higher than 50%, respectively. This phenomenon will also happen in a packed product though the rate at which it occurs depends on the water vapor permeability of the package film; the lower the water vapor transmission rate of the film, the slower the rate of water loss/gain.

**Figure 1:**
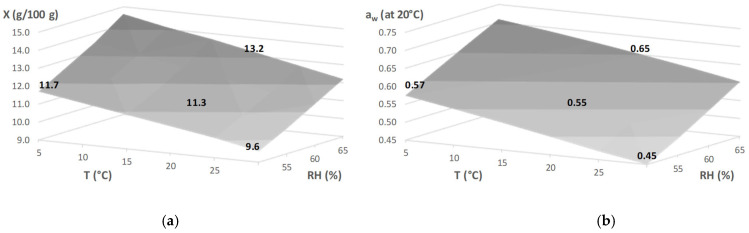
Water content (g/100 g, wet basis) of pasta prepared with 100% durum wheat depending on temperature and relative humidity (**a**) and water activity measured at 20 °C of the same pasta after reaching of equilibrium at the indicated temperature and humidity conditions (**b**). Calculations based on the desorption curves of De Temmerman et al. [16].

Therefore, the water content of the product suffers changes when exposed to varying conditions of temperature and a relative humidity of storage but can be predicted if the sorption isotherms of the product are known. De Temmerman et al. [16] obtained isotherms from pasta prepared with 100% durum wheat at temperatures between 20 and 80 °C. With the data published in that work, and by using the Oswin model and the respective parameters (Section 2.6), it was possible to predict the equilibrium water content for durum wheat pasta at the storage conditions used in our work (Figure 1a). The samples at 20 °C-65% RH tend to obtain the highest water content (13.2% wet basis) and samples stored at 30 °C-50% RH tend to obtain the lowest (9.6%). According to the conditions used in this work (similar to the water vapor permeability of the film), the area of the film used in the packages, and the mass of pasta per package, in 6 months all samples would be in equilibrium with the storage conditions. Moreover, if the water activity of the stored pasta at each condition was measured at 20 °C, again using De Temmerman et al. [16] isotherms, the water activities measured would be those presented in Figure 1b. For example, it can be predicted that pasta stored at 5 °C-50% RH reaches equilibrium at a water activity of 0.50 at 5 °C, but the water activity of the same pasta measured at 20 °C is 0.58.

**Figure 2:**
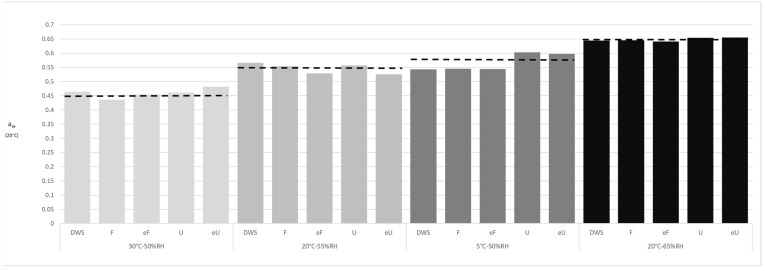
Water activity of pastas after 6 months stored at different temperature and relative humidity conditions at storage. Dashed line indicates the estimated water activity at 20 °C of samples stored at the temperature and relative humidity indicated.

In this work, the water activity of all samples after 6 months of storage was measured at 20 °C (Figure 2) which enabled the extrapolation of the water content and for comparisons to be made: the higher the water activity, the higher the water content for the same pasta (CTRL, U, eU, F or eF). The water activity values varied between 0.44 for *Fucus vesiculosus* at 30 °C-50% RH and 0.66 for *Ulva rigida* at 20 °C-65% RH. For each condition of temperature and relative humidity studied, the different pastas presented a similar water activity; approximately the expected water activity after equilibrium with the ambient air predicted from the isotherms of De Temmerman et al. [16] (Figure 1b) with slight differences between the seaweed used and whether it was used as raw flour or extract flour. Thus, all samples were close to equilibrium, allowing us to study the effect of this parameter on the pasta storage stability. The fact that the isotherms for the seaweed-enriched pastas were not available in the literature prevented the prediction of the water content of these samples. However, since the maximum content of seaweeds or their extracts was 1%, only small differences compared to the durum wheat pasta were expected.

Samples stored at 30 °C-50% RH were closer to equilibrium than those exposed to other conditions. This was understandable since the water transfer rate through the plastic film was proportional to the temperature, due to the higher permeabilities and vapor pressures verified at higher temperatures [17]. Note that, even though the pasta would be packaged with a lower water vapor permeability film than the one used in this work, water transfer could always occur through the film until an equilibrium with the environment was reached, provided that the product was stored for a sufficient time for this to occur.

There is a lack of criteria for estimating the shelf life of dried pasta. Some European Union countries debated whether to make pasta exempt from the obligation to have a best before date due to the long shelf life of dried pasta products [18]. To the authors’ knowledge, only the Food Safety and Standards Authority of India [19] established a criterion for the shelf life of durum wheat pasta, that consists of a maximum acceptable water content of 12.5%. According to this criterion, three of the storage conditions studied in this work led to lower water contents and only one condition led to a water content higher than 12.5%: the condition of 20 °C-65% RH. However, the water activity at this condition (0.65) (Figure 2) was still well below the limit above which concerns on microbial growth are usually justified [20].

**Figure 3:**
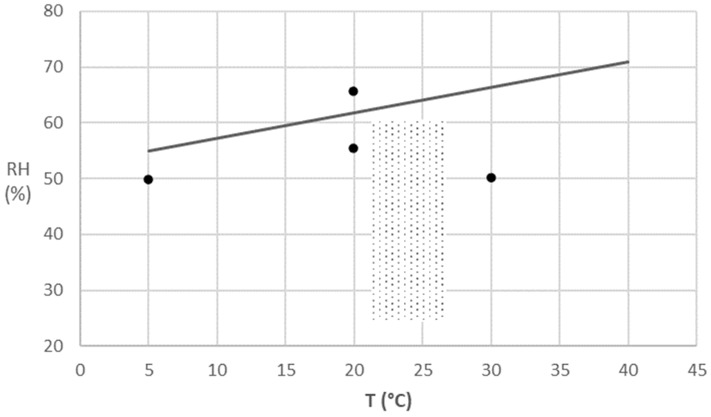
Temperature dependence of maximum relative humidity (―) to achieve a water content of 12.5% (wet basis) on durum wheat pasta based on the desorption curves of De Temmerman et al. [16], with indication of CEN 15251–2007 comfort standards for category II buildings (gray area) and conditions studied in this work (•).

Nevertheless, if a maximum water content of pasta was considered as a criterion for the end of its shelf life, to avoid reaching this limit, the maximum relative humidity (water activity) at each temperature which pasta must be stored, could be estimated. Figure 3 presents this limit (straight line) estimated based on the desorption curves of De Temmerman et al. [16] for ranges of temperature and relative humidity. The limit of relative humidity varies linearly between 5 °C-55% RH and 40 °C-71% RH, at a change ratio of 0.454% RH/ °C. If storage conditions are kept below these limits (at a higher temperature or a lower relative humidity), it is possible to ensure a long shelf life, if no other criterion apart from the water content is used. This will certainly be the case of packaged pasta kept in storehouses which follow the recommended comfort standards for category II buildings (a normal level of the expected conditions in new buildings and renovations) of the European standard CEN 15251-2007 [21]. These standards outline temperature and relative humidity values which stay below certain limits in order to achieve the water content limit defined by FSSAI (Figure 3).

If packaged pasta is subject to transport and storage in retail at environmental conditions of temperature and relative humidity that cause it to absorb a high amount of water, it is possible to determine the evolution of the water content over time by using typical mass transfer models [22] and predict when a maximum water content will be reached, and thus determine its shelf life.

### 3.2. Cooking Quality

The cooking quality of pasta can be assessed by several indicators such as weight gain, cooking loss, and textural properties [15] measured at the optimum cooking time (OCT). This is the time needed to completely gelatinize the starch present in pasta at a boiling temperature. The OCT was similar between semolina and pasta extracts (see Section 2.5) but increased when the seaweed flours were incorporated, with the exception Ulva extract (eU). This result was observed before and after the storage duration and independently of settled conditions. The extract ingredients may differ in fiber content from the seaweed flour. A higher fiber content demands more water for dough formation since both the fiber and gluten-protein matrix compete for water [23]. Moreover, an increase in fiber content may induce longer cooking times because it forms a barrier that delays water uptake and starch hydration. This result was observed in other studies [24,25] but was, however, controversial among literature studies focusing on pasta enrichment [26].

Pasta cooking performance was evaluated as cooking loss (solids loss), weight gain and firmness after cooking, and is presented in Table 2. These parameters were usually related between each other and provided a glimpse if too many nutrients were lost to the cooking water and if the pasta became too soft and sticky in the mouth [27]. Pearson correlations between the same parameters are presented in Table 3. 

#### 3.2.1. Influence of Storage Conditions over Time

After 6 months, no significant differences were observed in the cooking loss of all pastas and, regarding weight gain, significant differences were only observed for *Ulva rigida*-enriched pasta, with no clear trend with each water activity for the conditions studied. 

The storage conditions significantly affected the firmness of the pasta after cooking (*p* < 0.01 for all pastas). For durum wheat pasta, firmness increased when stored at 5 °C, was maintained when stored at 20 °C, and decreased when stored at 30 °C. For the other pastas, cooked firmness was consistently lower for pasta after 6 months in most of the storage conditions and for almost all enriched pasta studied, with exceptions observed for *F. vesiculosus* extract and *U. rigida* at 20 °C-55% HR. Replacing durum wheat semolina with seaweeds or extracts, even at a 1% level, may affect the gluten–starch matrix which confers cohesion and structure to pasta [24]. For example, Gallo et al. [28] described the influence of fibers on water mobility at molecular level in commercial spaghetti, resulting in structural loss after cooking. This structure alteration may be only slightly perceptible after the pasta confection but with a long storage time the weakening of bonds may increase and become more evident.

Similarly, Kaur et al. [29] observed that the cooking quality of fiber-enriched pasta remained constant during storage. In another study, Duszkiewicz-Reinhard et al. [10] observed lower cooking losses during storage for 6 months at 23 °C (no relative humidity was recorded) of legume and protein concentrates-enriched pasta but did not observe any change in the weight gain and firmness of raw pasta. It seems that dried pasta of any composition has a stability that prevents damage due to low water activities. Even at an extreme environmental condition of 38 °C-90%RH, pasta made exclusively with millet flour showed no changes in cooking loss and cooking firmness during 4 months of storage [30].

#### 3.2.2. Correlations between Cooking Quality Parameters

Weight gain and cooking loss follow a similar trend and, in fact, are highly correlated (r = 0.478, *p* < 0.01) (Table 3). The higher loss in seaweed flours-based pasta was most likely due to their higher fiber content, observed in pasta with no storage [24]. Fiber interferes with the gluten network in pasta facilitating the leaching out of components. The same reason may justify the higher mass gain of these pastas. 

Particularly relevant were the significant correlations of the texture parameters with the water activity at 20 °C for pastas after 6 months of storage. The correlation was negative meaning that a higher water content of packaged pastas after storage resulted in a lower firmness, either of raw (r = −0.296, *p* < 0.05) or of cooked (r = −0.518, *p* < 0.01) pasta. The decrease in the firmness of raw pasta may be explained by the higher hydration of polymers of protein, starch and fiber at the dried stage which led to less rigid pasta and resulted in a pasta which broke more easily. This effect was somehow transferred to the cooked pasta texture. This may be because a higher water activity during storage could cause a series of reactions, such as hydrolysis at a higher rate for a higher water content, which would give rise to smaller polymer chains that would be lost to the cooking water more easily. An additional explanation could be that when the water is absorbed by the packed pasta, the greater expansion of the polymers of the pasta may give rise to a larger area per volume, leading to a greater hydration during cooking, which in turn would cause a higher loss of solids. To avoid these changes, the recommended storage conditions are those that resulted in a lower water activity, achieved at lower environmental relative humidity and high temperature.

### 3.3. Sensory Analysis

The sensory acceptance is the main criteria of the industrial manufacturer to determine the end of the shelf life for food products in the absence of specific criteria for foods imposed by legislation. In this work, a global difference to the control was assessed for 2, 4 and 6 months of storage, as the main criteria to determine the shelf life. A reversed design was used in this study to test the influence of storage time and the environmental conditions of temperature–humidity on pasta. Therefore, all samples were available at the same time enabling a more accurate comparison. Additionally, it allowed for the measurement of the global sensory difference to the control instead of measuring the overall quality. 

**Figure 4:**
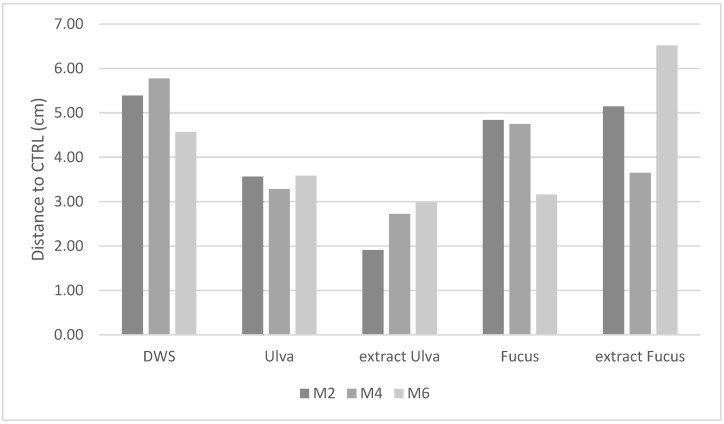
Difference to control between the pastas assessed over time (M2: month2; M4: month 4; M6: month 6) at each environmental condition studied.

Differences to control samples did not present significant values between each studied parameter (Figure 4). The data in Figure 4 represent the average value given among tasters for each considered month. Comparison to the control pasta showed that storage conditions did not alter the organoleptic properties of the durum wheat and seaweeds pasta over time (*p* > 0.05), but they did seem to depend on the type of seaweed enrichment. Nevertheless, changes over time were expected since the volatile compounds of seaweeds suffered changes during the storage time [31]. 

Overall, the differences between stored pastas are greater in the control pasta, followed by the Fucus pastas (flour and extract) and then the Ulva pastas (flour and extract). A considerable variability between the panelists’ ratings was observed, where some were usually bolder and others more conservative, inducing a high standard deviation of the obtained results. It seems that the sensory panel detected better differences in standard durum wheat pasta, for which organoleptic properties were more familiar with, compared to seaweeds enriched pasta. No observations of the unacceptable quality of pasta were made by the panelists, and thus all pastas were evaluated as acceptable for consumption after 6 months of storage in every condition studied.

In the literature, studies differ in the organoleptic modifications and overall acceptance of supplemented pasta after several months of storage. Duszkiewicz-Reinhard et al. [10] did not observe any change in the color, mouthfeel, external appearance, and general acceptability during storage for 6 months at 23 °C. Additionally, Verardo et al. [11] related a shelf life comparable to control pasta in functional spaghetti. In contrast, Sharma et al. [32] observed a decline in the overall acceptability scores of supplemented pastas with time, accentuated after the third month. The same authors related that a higher temperature (40 vs. 25 °C) and the type of packaging influenced the sensory properties of stored pasta. In millet pomace-based functional pasta, Gull et al. [30] reported that the overall acceptability scores remained good after four months of storage at 38 °C-90% RH. The variability in methods, supplemented ingredients, storage conditions, package film and shelf-life duration may induce these reported differences. 

## 4. Conclusions

Pasta is a dry product which remains very stable over time. Water activity was shown to be the storage parameter that best predicted the influence of the storage conditions on the quality parameters of pasta. A higher water activity of storage led to higher cooking loss and weight gain, and a lower firmness of cooked pasta. Very long storage durations evidenced a deterioration in the overall pasta quality. However, the water activity of storage would never reach values which limited the shelf life of pastas if normal temperature and humidity conditions in buildings (for example, according to CEN standards) were met. In this work, pasta stored in the appropriate conditions in warehouses did not suffer variations in their sensory properties compared to the control conditions (5 °C-50% RH). The use of only 1% of macroalgae or its extracts was found to be adequate, without influencing or modifying the sensory characteristics of the pasta samples during the storage period. These results were an indication that the shelf lives of these enriched pastas can be much longer than the validity times of the commercial pastas, from 1.5 to 2 years.

This study was confined only to a few quality factors which underwent changes during storage, and it was not possible to draw a conclusion regarding which limiting factor and its magnitude determined the limit of the lifespan of seaweed-enriched pasta. It should also be noted that the determination of the shelf life of pasta depended on the criteria of the producer since there were no established scientific limits (microbiological or other limits) for this type of products, except in the rare case where national laws specified such a limit.

## Figures and Tables

**Table 1:** Conditions and duration of pasta storage trial (reverse model).

Month	5 °C-50% RH	20 °C-55% RH			20 °C-65% RH			30 °C-50% RH		
**1**	*			*			*			*
**2**	*			*			*			*
**3**	*		*	*		*	*		*	*
**4**	*		*	*		*	*		*	*
**5**	*	*	*	*	*	*	*	*	*	*
**6**	*	*	*	*	*	*	*	*	*	*
**M#**	M6	M2	M4	M6	M2	M4	M6	M2	M4	M6

Notes: * This symbol indicates the month at which the samples were stored in the conditions identified at the top of the column. In the other months, samples were kept at 5 °C-50% RH. M#—# is the number of months at the conditions identified on the top of the column.

**Table 2:** Physicochemical parameters of pastas after preparation (fresh) and after storage for 6 months at four environmental conditions.

Pasta	Firmness Raw ×10^3^ (N)	Firmness Cooked (N)	Weight Gain (g/g)	Cooking Loss (g/100 g)
Durum wheat				
Fresh	2.81 ± 0.86	5.36 ± 0.07 ^b^	2.50 ± 0.04	4.26 ± 0.17
5 °C-50% RH	2.80 ± 1.08	6.70 ± 0.32 ^c^	2.55 ± 0.15	3.93 ± 0.66
20 °C-55% RH	2.16 ± 0.96	5.46 ± 0.43 ^b^	2.43 ± 0.03	3.40 ± 0.05
20 °C-65% RH	2.39 ± 0.79	4.89 ± 0.49 ^ab^	2.87 ± 0.14	3.54 ± 0.22
30 °C-50% RH	1.81 ± 0.90	4.36 ± 0.73 ^a^	3.25 ± 0.22	4.29 ± 0.24
	*p* = 0.163	*p* = 0.000	*p* = 0.094 *	*p* = 0.204 *
*F. vesiculosus*				
Fresh	2.21 ± 1.12 ^b^	6.10 ± 0.70 ^b^	2.64 ± 0.03	4.89 ± 0.14
5 °C-50% RH	1.09 ± 0.77 ^ab^	3.72 ± 0.27 ^a^	3.39 ± 0.05	5.68 ± 0.55
20 °C-55% RH	0.83 ± 0.27 ^ab^	3.96 ± 0.27 ^a^	2.69 ± 0.08	5.97 ± 0.25
20 °C-65% RH	0.55± 0.26 ^a^	3.60 ± 0.46 ^a^	3.48 ± 0.40	5.71 ± 0.06
30 °C-50% RH	1.31 ± 0.76 ^ab^	4.28 ± 0.12 ^a^	2.69 ± 0.05	5.27 ± 0.05
	*p* = 0.017 *	*p* = 0.000 *	*p* = 0.112 *	*p* = 0.160 *
*F. vesiculosus* extract				
Fresh	2.05 ± 1.05	4.90 ± 0.57 ^b^	2.95 ± 0.23	5.07 ± 0.45
5 °C-50% RH	1.58 ± 0.06	4.91 ± 0.49 ^b^	2.91 ± 0.36	5.24 ± 0.01
20 °C-55% RH	1.19 ± 0.38	4.87 ± 0.49 ^b^	2.98 ± 0.03	5.08 ± 0.11
20 °C-65% RH	1.20 ± 0.46	3.24 ± 0.36 ^a^	3.03 ± 0.58	4.73 ± 0.21
30 °C-50% RH	1.36 ± 0.54	3.22 ± 0.18 ^a^	2.80 ± 0.13	5.34 ± 0.16
	*p* = 0.116 *	*p* = 0.000 *	*p* = 0.861 *	*p* = 0.114
*Ulva rigida*				
Fresh	1.56 ± 1.36	5.02 ± 0.75 ^c^	2.60 ± 0.04 ^a^	4.23 ± 0.08
5 °C-50% RH	1.43 ± 0.04	3.61 ± 0.10 ^b^	3.17 ± 0.10 ^bc^	4.62 ± 0.41
20 °C-55% RH	1.67 ± 0.73	4.92 ± 0.21 ^c^	2.88 ± 0.17 ^ab^	5.11 ± 0.06
20 °C-65% RH	1.75 ± 0.45	2.99 ± 0.32 ^a^	3.44 ± 0.22 ^c^	4.88 ± 0.42
30 °C-50% RH	1.51 ± 0.42	3.54 ± 0.13 ^b^	2.68 ± 0.03 ^a^	4.23 ± 0.71
	*p* = 0.553 *	*p* = 0.000	*p* = 0.047 *	*p* = 0.252 *
*Ulva rigida* extract				
Fresh	1.01 ± 0.99 ^a^	5.06 ± 0.04 ^c^	3.08 ± 0.09	4.83 ± 0.13
5 °C-50% RH	2.55 ± 0.68 ^b^	2.99 ± 0.17 ^a^	2.87 ± 0.26	4.14 ± 1.30
20 °C-55% RH	2.06 ± 0.84 ^b^	4.61 ± 0.18 ^b^	2.91 ± 0.05	4.45 ± 0.49
20 °C-65% RH	1.76 ± 0.65 ^ab^	3.29 ± 0.23 ^a^	2.17 ± 0.03	3.57 ± 0.89
30 °C50% RH	2.46 ± 0.52 ^b^	3.09 ± 0.15 ^a^	2.76 ± 0.39	4.37 ± 0.07
	*p* = 0.001	*p* = 0.000 *	*p* = 0.211 *	*p* = 0.249 *

Significant differences at *p* < 0.05 between the same pasta when fresh and stored in different storage conditions are noted with different letters. * symbol refers to Kruskal–Wallis test *p* value.

**Table 3:** Pearson correlation of cooking quality parameters of pastas at 6 months.

	Firmness Cooked	Mass Gain	Cooking Loss	aw
Firmness raw	0.196 **	0.034	−0.088	−0.296 *
Firmness cooked		−0.022	−0.143	−0.518 **
Mass gain			0.478 **	0.000
Cooking loss				0.234

* *p* < 0.05; ** *p* < 0.01.

## Data Availability

Not applicable.
